# Extrinsic and intrinsic drivers of prevalence and abundance of hard-bodied ticks (Acari: Ixodidae) in one-humped camel (*Camelus dromedarius*)

**DOI:** 10.1016/j.parepi.2024.e00387

**Published:** 2024-10-21

**Authors:** Rachid Chaibi, Nora Mimoune, Farouk Benaceur, Latifa Stambouli, Lamine Hamida, Rabah Khedim, Radhwane Saidi, Mohammed Hocine Benaissa, Hicham Gouzi, Souad Neffar, Haroun Chenchouni

**Affiliations:** a Department of Biology, Faculty of Sciences, University of Laghouat, 03000 Laghouat, Algeria; b Laboratory of Biological and Agronomic Sciences ‘LBAS’, University of Laghouat, 03000 Laghouat, Algeria; c Animal Health and Production Laboratory, Higher National Veterinary School, Algiers, Algeria; d Institute of Veterinary Sciences, LBRA, University of Blida 1, 09000 Blida, Algeria; e Aflou University Center, 03001 Aflou, Laghouat, Algeria; f Department of Agronomy, Faculty of Sciences, University of Laghouat, 03000 Laghouat, Algeria; g Scientific and Technical Research Centre for Arid Areas (CRSTRA), Biophysical Station, 30010 Nezla, Touggourt, Algeria; h Department of Nature and Life Sciences, Faculty of Exact Sciences and Nature and Life Sciences, University of Tebessa, 12002 Tebessa, Algeria; i Laboratory “Water and Environment”, University of Tebessa, 12002 Tebessa, Algeria; j Laboratory of Algerian Forests and Climate Change 'LAFCC', Higher National School of Forests, 40000 Khenchela, Algeria; k Laboratory of Natural Resources and Management of Sensitive Environments ‘RNAMS’, University of Oum-El-Bouaghi, 04000 Oum-El-Bouaghi, Algeria

**Keywords:** Camel farming, Dromedary health, Ixodid tick species, Tick infestation, Parasite prevalence, Parasite load, Tick ecology, Tick control, Tick-borne diseases, Tick abundance, Ectoparasite epidemiology

## Abstract

**Background:**

Ticks are ectoparasites and can be vectors of a wide range of pathogens, posing significant health risks to livestock. In the Sahara Desert of Algeria, particularly among one-humped camels (*Camelus dromedarius*), there is a need to better understand the factors influencing tick infestation patterns to improve livestock management and health outcomes.

**Objectives:**

This study aimed to investigate the prevalence, intensity, and abundance of hard-bodied ticks (Acari: Ixodidae) among dromedaries, examining both intrinsic factors (sex, age, coat color) and extrinsic variables (farming systems, vegetation types, climate zones, and elevation) that might influence tick infestation in this region.

**Methods:**

Ticks were collected from 286 dromedaries across nine sites in the pre-Saharan regions of Algeria, with elevations ranging from 736 m to 980 m. The sampled camels, which ranged in age from 6 days to 21 years, were examined for tick infestations. The ticks were identified through macroscopic and microscopic methods, and their abundance was analyzed in relation to the camels' characteristics and environmental factors. Three breeding systems were recognized: extensive, intensive, and mixed.

**Results:**

A total of 980 ticks were collected, with *Hyalomma dromedarii* Koch, 1844 being the most abundant species (553 specimens), followed by *Hyalomma impeltatum* Schulze & Schlottke, 1930 (393 specimens), and *Hyalomma excavatum* Koch, 1844 (34 specimens). *H. dromedarii* showed a preference for parasitizing brown-coated dromedaries and exhibited significantly higher infestation levels during spring (*p* < 0.001). No significant association was observed between tick infestation and the camels' age or sex (*p* > 0.05). However, the farming system had a significant impact on tick abundance, with extensive and mixed systems showing higher tick burdens compared to intensive systems (*p* < 0.01). Additionally, the vegetation type, climate zone, and foraging habitat elevation were found to significantly influence tick densities and prevalence.

**Conclusion:**

This study provides essential insights into the tick infestation dynamics in dromedaries in drylands of Algeria. It highlights the influence of coat color, seasonality, and farming practices on tick burden, with brown-coated camels being more susceptible during the spring. The findings underline the importance of considering both intrinsic and extrinsic factors when developing effective tick control strategies, especially for camels raised in extensive or mixed farming systems in diverse arid rangelands. Future research should expand the scope to cover other arid regions in North Africa for a comprehensive understanding of tick-host dynamics.

## Highlights

• Prevalence and intensity of ixodid ticks in *Camelus dromedarius* were studied.
• 980 ticks of the genus *Hyalomma* were found on 286 dromedaries in drylands at N. Africa.
• 3 species were identified: *H. dromedarii* (56.4 %), *H. impeltatum* (40.1 %), *H. excavatum* (3.5 %).
• Tick densities were high in spring (1.89 ± 0.14) and summer (1.44 ± 0.15), and low in winter (0.03 ± 0.02).
• Parasite prevalence and intensity was affected by both intrinsic and extrinsic variables.

## Graphical abstract

**Figure:**
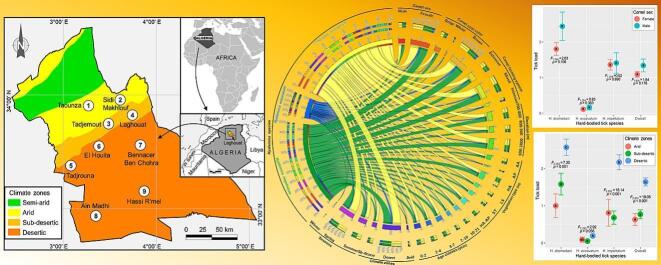

## 1 Introduction

The dromedary (*Camelus dromedarius*) represents the animal without which the great nomadic civilizations could never have existed (Benaissa et al., 2012); it expresses remarkable capacities of adaptation allowing it to make the best use of the resources available in Saharan ecosystems (Benaissa et al., 2012; Faye, 2020; Padalino and Faye, 2024). In North African countries, camel farming represents a central activity in the steppe and desert pastoral areas. For the same region, the total camel population is also reported to have declined over the past 50 years, from 1,031,000 head to 879,000 head in 2011. Although, Algeria - an exceptional ecological entity including various ecosystems - is home to a large number of plant and animal species (Mouane et al., 2024), there is a single species of camel (*Camelus dromedarius*) in the country (Bouhous et al., 2008; Saidi et al., 2022). The number of camels in Algeria ranks 19th in the world (1.32 % of the world livestock, 2 % of the Arab camel population, and 13 % of the individuals of the Maghreb region) (Harek et al., 2017). The Ministry of Agriculture and Rural Development in Algeria identified nearly 340,140 camels including 200,284 females in 2012. It was conducted according to different breeding systems with predominance of the extensive farming systems is arid and semi-arid rangelands.

Contemporary parasitic ecology is a rapidly advancing field, primarily because ecologists are increasingly considering the possible role parasites play in regulating host population dynamics and their impact on ecosystem equilibrium and functionality (Estrada-Peña and de la Fuente, 2014). In broader context, parasitism stands as merely one conceivable manifestation of symbiotic interaction between two organisms (Wood and Johnson, 2015). Ticks denote hematophagous arthropods within the Chelicerata sub-phylum and Arachnida class. These ectoparasites engender a significant potential as vectors of human and animal diseases. Remarkably, they exhibit prodigious longevity, spanning up to a decade, throughout which they possess the capacity to exploit numerous vertebrate hosts (Claudine, 2007; Socolovschi et al., 2008).

After mosquitoes, ticks are serious vectors of viruses that concern both human and veterinary health (Yu et al., 2015). Ticks represent a distinct group of ectoparasites characterized by specific host preferences, ecological niches, and infection biology (Sonenshine, 1991; Claudine, 2007; Estrada-Peña and de la Fuente, 2014; Viglietta et a., 2021). They encompassing nearly 869 species, which includes hard ticks (Ixodidae) and soft ticks (Argasidae). They are found all over the world, in cold- and hot-desert areas as well as in lowland and highland regions (Perveen et al., 2021), and they can transmit a wide variety of parasites, virus and bacteria (Jongejan and Uilenberg, 2004; Goodman et al., 2005; Geraci et al., 2007; Chadi et al., 2024; Ergunay et al., 2024). In particular, they can transmit various tick-borne diseases, including Lyme disease, anaplasmosis, babesiosis, Tularemia, and Ehrlichiosis, leading to significant health issues in their hosts (Schmidt and Roberts, 1977; Sonenshine, 1991; Socolovschi et al., 2008). In recent years, an emergence of new tick-borne diseases has been recorded, as well as an increase in the rate of existing diseases with a change in their epidemiology: prevalence, pathogenicity, and geographic distribution (Shaw et al., 2001; Beugnet and Marie, 2009). The variety of host species can vary greatly depending on tick species. Ixodid ticks generally appear on up to three hosts throughout their life cycle; some species are one-host or two-host, while others are three-host ticks (Nicholson et al., 2019). In the larval stage, ixodid ticks feed on two distinct host species, while in the nymphal and adult stages, they feed on various types of mammals (Carpenter et al., 1996). Furthermore, tick infestation depends on several environmental and host factors (Kovats et al., 2001). They feed more quickly in warm temperatures, with repeated infestations. Low temperatures result in an increase in feeding duration and lead to an increase in engorgement weight (Norval, 1978).

Effectively addressing diverse variables is crucial for improving camel management in dryland regions. Investigating intrinsic traits of dromedaries in relation to tick burden is of particular importance, as it reveals distinct behaviors and immunological responses within host populations that influence tick dynamics (Dioli et al., 2001; Bouhous et al., 2008; Moshaverinia and Moghaddas, 2015; Seddik et al., 2016; Rehman et al., 2017). This knowledge guides targeted, age-specific tick control strategies, and therefore enriching comprehensive livestock health management (Estrada-Peña and de la Fuente, 2014; Sazmand et al., 2019; Perveen et al., 2021; Padalino and Faye, 2024). Equally essential is comprehending how camel farming systems interact with tick prevalence and parasitism patterns. The human intervention, grazing patterns, and animal densities intrinsic to these systems are still not well explored to determine their impacts on tick exposure and infestation dynamics (Diuk-Wasser et al., 2021). Additionally, investigating environmental extrinsic factors such as spatio-temporal changes in the characteristics of foraging rangelands assumes significance to understand health status of dromedary production system (Temple and Manteca, 2020). Varied ecosystems offer diverse microclimates and foraging habitats that mold parasite distribution patterns and prevalence (Kabbout et al., 2016; Attir et al., 2024). Elevation gradients and climatic zones further modulate environmental conditions affecting tick activity, host behaviors, and parasite-host interactions (Jore et al., 2011; Dantas-Torres, 2015; Domșa et al., 2016). A holistic grasp of these factors underpins the tailoring of tick control strategies to specific environments, culminating in an enhanced paradigm for camel health and management within arid regions.

Although studies from Algeria on the prevalence and abundance of ticks in dromedary camels are relatively numerous, they have largely remained localized and focused primarily on either tick species identification or parasite indices (Bouhous et al., 2008; Ouchene-Khelifi et al., 2020; Lakehal et al., 2021; Saidi et al., 2022; Betatache et al., 2024; Attir et al., 2024). These earlier works, while valuable, tend to lack a comprehensive examination of the factors influencing tick infestation beyond basic epidemiological assessments. In contrast, this study distinguishes itself by addressing a broader range of variables, both intrinsic (related to the host) and extrinsic (environmental and habitat-related), that may impact parasite load. Specifically, this research goes beyond mere tick identification to explore how factors such as camel coat color, age, sex, and farming systems interact with environmental conditions like rangeland vegetation type, elevation, and seasonal changes in the Sahara Desert. This examined these complex interactions and provided a more holistic understanding of the factors influencing tick dynamics in dromedaries to offer practical insights into camel farming systems in arid environments. This approach not only enhances the understanding of tick ecology in this region but also contributes to improved parasite management and camel health practices across the Algerian Sahara (Padalino and Faye, 2024).

Because of the importance of parasitic arthropods, either by their abundance and their direct predatory action, by the transmitted pathogens (protozoa, bacteria and viruses) or their own toxins (Sonenshine, 1991; Yu et al., 2015; Kabbout et al., 2016; Perveen et al., 2021; Chadi et al., 2024), the following objectives were designed: (i) identify and quantify the tick species in camelid population raised in different climatic zones of a hot arid region in North Africa (Laghouat, Algeria), (ii) explore the relationships that may exist between the parasite load and several extrinsic (associated to the environment and foraging habitats) and intrinsic (i.e. associated to the host) variables related to the camel farming systems in the Sahara Desert of North Africa. A deep understanding of the complex interaction of intrinsic and extrinsic variables in camel management and tick control within drylands is imperative for several key reasons. Firstly, understanding the dynamics of camel sex ratios and their susceptibility to tick infestation is critical for targeted management strategies. We expect that dealing with variations in tick loads between male and female camels could reflect underlying physiological, behavioral, or immunological differences that influence infestation rates and pathogen transmission.

## 2 Materials and methods

### 2.1 Study area

**Fig. 1:**
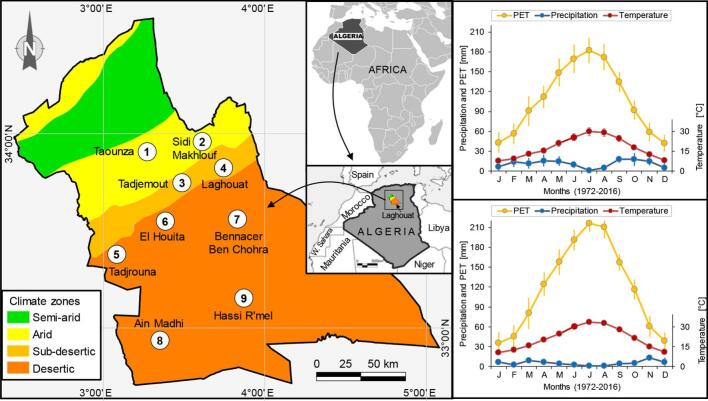
Location of study sites (solid white circles, description in Table 1) on the map of bioclimate zones of the region of Laghouat in Algeria. Plots on the left represent Gaussen's ombrothermic diagrams applied for the arid and sub-desertic zones (upper plot) and desertic climate (bottom plot), with mean temperature (in °C), precipitation (in mm) and potential evapotranspiration ‘PET’ (in mm) are monthly averages for the period 1972–2016.

This study was conducted across nine sites, each associated with a different administrative city, namely Aïn Madhi, El Houita, Hassi R'Mel, Laghouat, Bennacer Ben Chohra, Sidi Makhlouf, Tadjmout, Tadjrouna, and Taounza in Algeria. These sites are spread across three distinct bioclimatic regions, namely arid, sub-Saharan, and Saharan (Fig. 1). Over an eight-month period, spanning from December 2016 to July 2017, all sites underwent multiple sampling sessions, with at least one monthly survey. The surveyed landscapes predominantly consisted of arid rangelands characterized by steppe vegetation (Merdas et al., 2021) and its characteristic species: halfah grass *Stipa tenacissima* (Syn. *Macrochloa tenacissima* (L.) Kunth), esparto grass *Lygeum spartum* Loefl. ex L., *Aristida pungens* Desf., *Astragalus armatus* Willd., and *Hammada articulata* (Moqu.) O.Bolos & Vigo.

The selection of Laghouat as the study area is justified by its distinctive landscape and prevalent livestock practices. Known for its primary focus on sheep and goat rearing in arid and semi-arid rangelands, Laghouat also incorporates camel keeping in both climatic regions. This dual presence of various livestock species, including camels, in a challenging environment makes Laghouat an ideal location for investigating the complexities of camel parasitism. This choice enables a comprehensive examination of the interplay between intrinsic and extrinsic factors influencing tick infestation patterns in dromedaries, providing valuable insights for local husbandry practices and broader ecological considerations.

### 2.2 Description, typology and sampling of one-humped camels

The study was approved by the Scientific Committee of the Faculty of Sciences of the University of Laghouat (Algeria). All procedures were carried out in accordance to the standards and regulations set by the Faculty's Ethics Committee. The study also followed the Animal Research: Reporting of In Vivo Experiments (ARRIVE) guidelines (Percie du Sert et al., 2020) and the U.K Animals Act 1986 guidelines (Hollands, 1986). The owners of the surveyed camels provided their verbal agreement for the tick samples to be collected.

In the nine sites selected, a total of 286 camels were conveniently examined. This samples size was determined to represent diverse conditions of the intrinsic and extrinsic variables. The studied population had an age range from a few days (6 days) to 21 years, whereas the sex ratio was in favor of females (M:F = 0.28), with 224 females (78 %) against 62 males (22 %). The camel population under investigation is distributed across sites characterized by elevations ranging from 736 m to 980 m. Three types of breeding systems were recognized in the studied region: extensive (based on camel mobility in pastures and rangelands, low inputs, and low market integration), intensive (based on feeding by irrigated feedstuffs, settlement, and market integration), and mixed. The 286 dromedaries examined have either beige/cream, white, or brown coat color (Table 1).

**Table 1:** Typological characterization of the different sites surveyed, with description of the camel farming sampled at the region of Laghouat in Algeria. The ID refers to the site codes described in Fig. 1.

ID	Sites	Elevation (m a.s.l.)	Dominant plant species	Camel farming information				
				Farming systems	Female	Male	Age range [Years]	Coat colors
1	Taounza	820	*Stipa tenacissima* (ST)	Intensive	11	4	1–12	BG, BR, WT
2	Sidi Makhlouf	924	*Astragalus armatus* (AA)	Mixed	24	8	1–12	BG, BR, WT
3	Tadjmout	843	*Astragalus armatus* (AA)	Mixed	26	5	1–20	BG, BR
4	Laghouat	789	*Aristida pungens* (AP)	Intensive	66	13	1–13	BG, BR, WT
5	Tadjrouna	916	HA + AP	Extensive	22	8	1–11	BR
6	El Houita	919	*Lygeum spartum* (LS)	Mixed	24	7	1–10	BG, BR
7	Bennacer Ben Chohra	736	*Hammada articulata* (HA)	Extensive	15	6	1–12	BR
8	Ain Madhi	983	*Stipa tenacissima* (TS)	Mixed	14	1	1–06	BG, BR, WT
9	Hassi R'mel	750	*Hammada articulata* (HA)	Mixed	23	10	1–12	BG, BR

(BG: beige, BR: brown, WT: white)

### 2.3 Collection and identification of ticks

With the animal in a standing position, the entire body of the animal was inspected with emphasis on the preferential sites of tick attachment, namely the sternal, inguinal and perineal regions. Using entomological forceps, ticks were manually collected from the host and were stored in hermetically sealed vials containing 70 % ethanol. Each vial was labelled with the sample number, site, date of collection and host code. The identification of adult tick stages was conducted in the laboratory through observation using a binocular magnifying glass. This observation focused on the morphological characteristics of specific parts of the tick's body, with particular attention to the rostrum, eyes, and festoons. These features were examined to determine the tick's genus, in accordance with the identification keys and guidelines described previously (Walker, 2003; Abdullah et al., 2016; Diaha-Kouame et al., 2020). These latter references report specific characteristics and the taxonomy of each *Hyalomma* tick species encountered in this study.

### 2.4 Data management and statistical analysis

Data collected during the study period were explored and summarized according to three intrinsic factors: sex ratio (camel sexes), camel coat colors (brown, beige or white), and camel age classes, and five extrinsic variables, i.e. either related to camel farming system (extensive, intensive or mixed) or the environment of the surveyed sites (steppe rangeland types, Table 1), elevation, seasons, and climate zones (arid, sub-desertic or desertic). The parasite indices (parasite prevalence, parasite abundance, and average parasite intensity) were determined for each variant of the eight above-mentioned variables. Parasite prevalence (*P%*) was defined as the ratio of the number of parasitized hosts (*HP*) with a given parasite species to the number of hosts examined (*N*): *P(%) = HP/N ×* 100. Parasite abundance (*AB*) was defined as the ratio of the total number of individuals of a parasite species (*Np*) to the total number of host individuals examined (*N*): *AB* = *Np/N*. Average parasite intensity (*IM*) was defined as the ratio of the total number of individuals of a parasite species (*Np*) in a sample of hosts to the number of infested hosts (*HP*) in the sample: *IM = Np/HP*.

The statistical analysis and graphics were done using the R software version 4.4.0 (R Core Team, 2024). To allow comparisons between variants of the intrinsic and extrinsic variables, the data of each tick species was summarized using descriptive statistics, such means and standard error of mean (SE). The variation of tick load of each species and all species combined ‘overall’ was tested using generalized linear models (GLM) at *p* < 0.05. Count data of ticks were fitted to a Poisson distribution and log link function after checking the equality of variance. Each tested GLM was summarized using likelihood-ratio test where Chi-square and *p*-value were used as test statistical outputs.

Furthermore, the variation of tick abundance per species and for all species combined was tested following the effects of all the intrinsic and extrinsic factors. Here, we used a negative binomial distribution and log link function in GLMs. The initial full GLM, encompassing all eight independent variables, underwent simplification through a step-wise backward-forward selection procedure. The final selection of the model with the best fit was based on the lowest Akaike information criterion (AIC) value. In this context, models with an AIC difference greater than 2 were considered significantly different.

## 3 Results

### 3.1 Parasitized camels and identified tick species

**Fig. 2:**
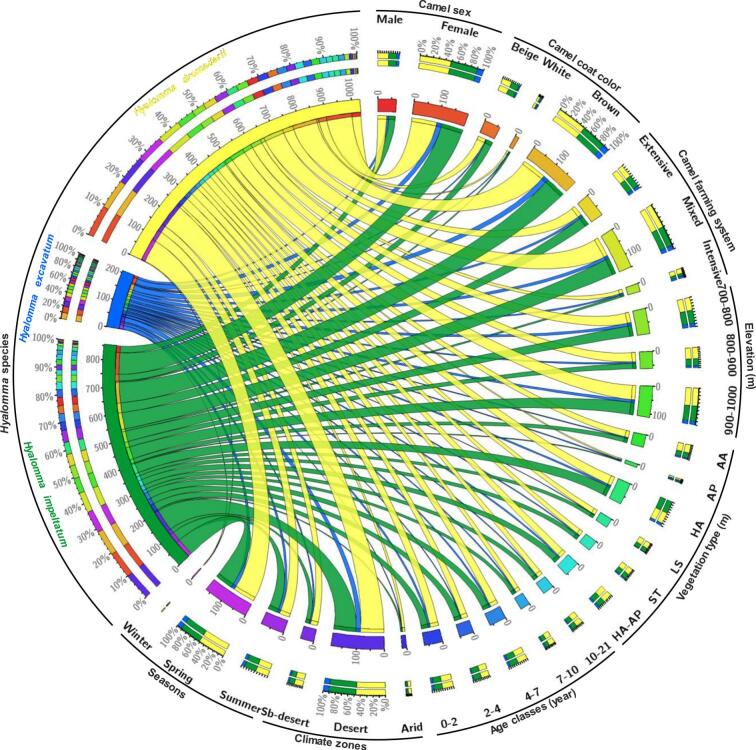
Chord diagram displaying the distribution of the total number of dromedaries parasitized by hard- ticks following different variables related to the host and its environment.

In the surveyed sites in Laghouat, 286 camels were examined, comprising 61 males (22 %) and 225 females (78 %) (Fig. 2). The host population had an age structure ranging from a few days to 21 years, while 50 % of the individuals aged between 2 and 7 years, with 50 % of the individuals of age between 0.1 and 4 years for males and between 2 and 7 years for females.

Parasitized camels with brown coats were of the highest proportion (57.3 %), followed by beige (35.7 %) and then white (7 %) coats. Ectoparasitism occurred mainly in mixed systems (50 %) compared to extensive (17 %) and intensive (33 %) farming systems. The infested camel populations occurred mostly in arid rangelands located at elevation ranging between 700 and 800 m (46.2 %) and 900–1000 m (37.8 %), with vegetation facies of *A. pungens* (27 %), *A. armatus* (22 %), *H. articulata* (18 %), then the rest of the rangelands included L. *spartum*, *S. tenacissima* and *H. articulata* + *A. pungens* (10 %). More than 80 % of these individuals were sampled in desert and sub-desert climate zones during spring and winter (Fig. 2).

Overall, the parasite infestation rate was 55.9 % (160/286), affecting 70.5 % of males and 53.3 % of females, of which 98 % were reared in extensive farming systems vs. 68.8 % and 15.9 % in mixed and intensive systems, respectively. The order of parasitized camels in regard to rangeland vegetation types was *H. articulata + A. pungens* (100 %), *H. articulata* (96.2 %), *S. tenacissima* (83 %), *L. spartum* (67.7 %), *A. armatus* (51.6 %) and then *A. pungens* (6.3 %). Out of the 286 examined camels, 178 individuals (62.7 %) were parasitized in the desert climatic region, against 21.3 % and 16 % under sub-desert and arid climatic conditions (Fig. 2). Under desert conditions, parasite prevalence was about 70 % of camels, whereas in sub-desert and arid climatic regions it was 24.5 % and 21.3 %, respectively (Table 2). The infestation rate peaked in summer (98 %) and spring (82 %) as compared to winter with 1.94 %. In addition, it was maximum (89 %) at elevation of 800–900 m. Half of the infested individuals belonged to the age category of 2 to 10 years. Camels with brown coats were the most infested with a proportion of 70 %, followed by beige and white phenotypes with 40 % and 35 %, respectively (Table. 2).

**Table 2:** Parasite indices (prevalence, intensity, and abundance) of hard-bodied ticks in one-humped camel according to intrinsic variables (i.e. host population and farming characteristics) extrinsic factors (i.e. environmental variables) in drylands of Algeria. (*N*: sample size (camels), *P%*: prevalence; *IM*: average intensity, *AB*: abundance); vegetation types: (AA: *Astragalus armatus*, AP: *Aristida pungens*, HA: *Hammada articulata*, LS: *Lygeum spartum*, ST: *Stipa tenacissima*).

Variables	Categories	*N*	*H. dromedarii*			*H. impeltatum*			*H. excavatum*			All species		
			*P%*	*IM*	*AB*	*P%*	*IM*	*AB*	*P%*	*IM*	*AB*	*P%*	*IM*	*AB*
Camel sex	Male	61	55.7	4.4	2.4	41.0	3.4	1.4	13.1	1.3	0.2	70.5	5.7	4.0
	Female	225	42.7	4.2	1.8	36.9	3.7	1.4	8.4	1.3	0.1	53.3	6.1	3.3
Camel coat color	Beige	102	29.4	5.2	1.5	24.5	4.2	1.0	3.9	1.3	0.0	40.2	6.5	2.6
	Brown	164	56.7	4.1	2.3	48.2	3.5	1.7	12.2	1.4	0.2	70.1	5.9	4.2
	White	20	35.0	2.0	0.7	20.0	3.8	0.8	15.0	0.3	0.1	35.0	4.3	1.5
Camel age class [year]	[0–2[	68	48.5	4.5	2.2	38.2	3.8	1.4	10.3	1.3	0.1	58.8	6.4	3.8
	[2–4[	70	35.7	3.6	1.3	34.3	4.1	1.4	4.3	1.3	0.1	50.0	5.5	2.7
	[4–7[	70	42.9	4.4	1.9	37.1	3.7	1.4	8.6	1.3	0.1	51.4	6.6	3.4
	[7–10[	40	45.0	4.0	1.8	32.5	2.8	0.9	10.0	2.0	0.2	55.0	5.3	2.9
	[10−21]	38	55.3	5.2	2.9	42.1	3.9	1.7	10.5	1.3	0.1	71.1	6.6	4.7
Camel farming system	Extensive	50	80.0	2.8	2.2	74.0	2.5	1.8	18.0	1.6	0.3	98.0	4.4	4.3
	Mixed	142	52.8	5.2	2.7	44.4	4.2	1.9	8.5	1.3	0.1	69.7	6.7	4.7
	Intensive	94	16.0	3.7	0.6	8.5	4.8	0.4	6.4	0.8	0.1	16.0	6.5	1.0
Steppe rangeland type	AA	63	39.7	6.6	2.6	25.4	4.7	1.2	6.3	1.3	0.1	49.2	7.9	3.9
	AP	79	6.3	1.6	0.1	3.8	0.0	0.0	5.1	0.3	0.0	6.3	1.8	0.1
	HA	53	66.0	4.4	2.9	69.8	3.8	2.6	13.2	1.3	0.2	96.2	5.9	5.7
	LS	31	61.3	5.0	3.1	41.9	3.5	1.5	6.5	1.5	0.1	67.7	6.8	4.6
	TS	30	63.3	4.3	2.7	53.3	5.1	2.7	13.3	1.5	0.2	83.3	6.7	5.6
	HA + AP	30	90.0	1.8	1.6	76.7	2.3	1.8	20.0	1.7	0.3	100.0	3.7	3.7
Site elevation a.s.l. [m]	[700–800[	132	30.3	4.1	1.2	30.3	3.5	1.1	8.3	0.9	0.1	42.4	5.6	2.4
	[800–900[	46	76.1	6.1	4.6	45.7	5.4	2.5	13.0	1.5	0.2	89.1	8.2	7.3
	[900–1000]	108	50.9	3.2	1.6	43.5	3.0	1.3	9.3	1.5	0.1	61.1	5.0	3.1
Seasons	Winter	103	1.9	4.0	0.1	0.0	0.0	0.0	1.0	1.0	0.0	1.9	4.5	0.1
	Spring	133	63.9	5.1	3.3	51.1	4.4	2.3	10.5	1.4	0.1	82.0	6.9	5.7
	Summer	50	80.0	2.8	2.2	74.0	2.5	1.8	18.0	1.6	0.3	98.0	4.4	4.3
Climate zones	Arid	47	21.3	4.7	1.0	10.6	7.6	0.8	4.3	2.0	0.1	21.3	8.9	1.9
	Sub-desertic	110	24.5	6.4	1.6	14.5	4.7	0.7	4.5	1.2	0.1	30.0	7.7	2.3
	Desertic	129	69.8	3.7	2.6	65.1	3.3	2.2	13.2	1.4	0.2	90.7	5.4	4.9
Total		286	44.4	4.4	1.9	36.7	3.7	1.4	8.4	1.4	0.1	55.9	6.1	3.4

**Fig. 3:**
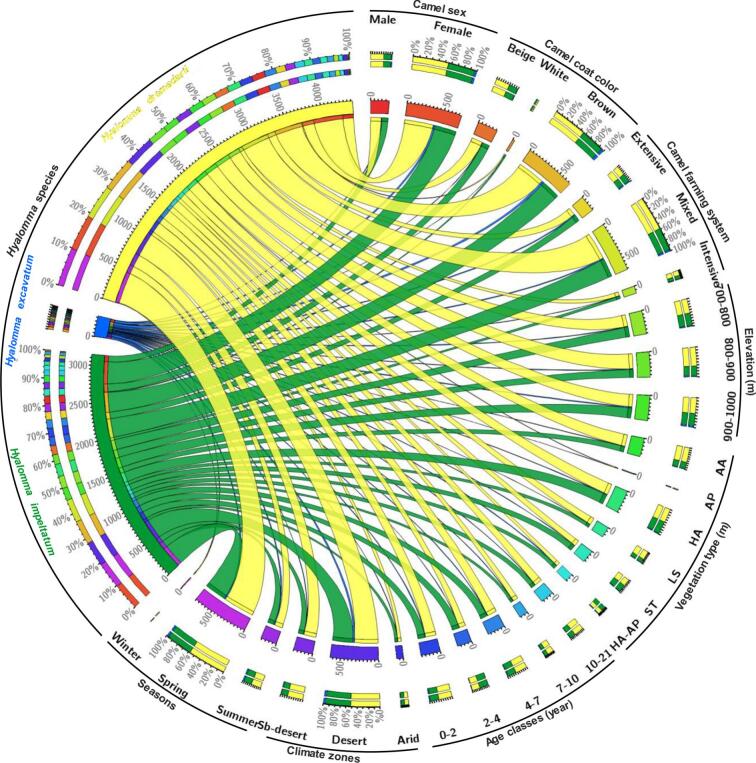
Chord diagram showing the distribution of the total numbers of tick species parasitizing dromedaries following different variables related to the host and its environment.

The total number of ticks collected was 980, where the most abundant tick was *Hyalomma dromedarii* Koch, 1844 (553 specimens, 56.4 % of the total) followed by *Hyalomma impeltatum* Schulze & Schlottke, 1930 (with 393 specimens, 40.1 %), while the least frequent tick species was *Hyalomma excavatum* Koch, 1844 (34 specimens, 3.5 % in total) (Fig. 3). This abundance fluctuated according to the intrinsic and extrinsic variables when considered separately. *Hyalomma dromedarii* predominated in both males and females with 60.8 % and 55 %, respectively, followed by *H. impeltatum* (35.1 % and 41.8, respectively) then *H. excavatum* (4.1 % and 3.3 %, respectively). Apart from a few rare exceptions, the species *H. dromedarii* infested up to 59 % of camels regardless of age, coat color, in the three camel farming systems, at different elevations, in different rangeland vegetation types, under the three types of climates during the three seasons, while the *H. excavatum* was least abundant revealing low values for each variable mentioned above (Fig. 3).

### 3.2 Intrinsic factors affecting dromedary ectoparasitism (univariate analysis)

#### 3.2.1 Number of ticks by camel sex

**Fig. 4:**
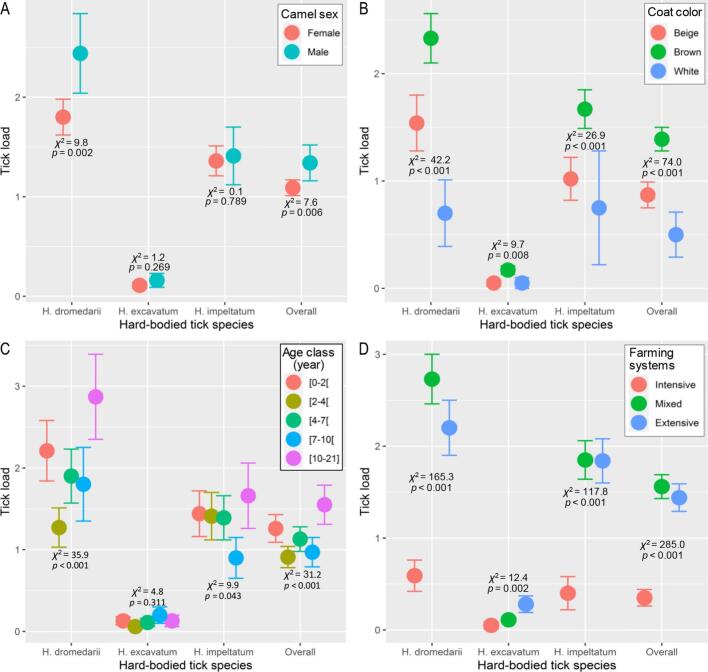
Plot of means (± standard errors, vertical error bars) of the parasite abundance of three hard-bodied tick species for the two sexes (A), coat colors (B), different age classes (C), and different farming systems (D) of the one-humped camel (*Camelus dromedarius*) in drylands of Algeria. The statistics *χ*^2^ and *p*-values are the summarized results of likelihood-ratio tests calculated for generalized linear model of each species separately and overall species.

Fig. 4 compares the number of each tick species encountered according to the sex of the dromedary. For *H. dromedarii*, tick load averaged 1.80 ± 0.18 (mean ± SE) in females vs. 2.44 ± 0.40 in males. This average was 0.11 ± 0.03 for *H. excavatum* in females and 0.16 ± 0.07 ticks in males. The mean density of *H. impeltatum* was 1.36 ± 0.15 for females and 1.41 ± 0.29 for males. For all species combined, female dromedaries recorded an average of 1.09 ± 0.08 ticks and males had 1.34 ± 0.18 ticks (Fig. 4A). These differences were statistically significant in *H. dromedarii* (GLM: *p* = 0.002) and for all species combined (*p* = 0.006).

For all tick species combined, the prevalence was 70.5 % in males and 53.3 % in females. The parasite intensity ranged from 5.7 to 6.1. The *H. dromedarii* species had a higher prevalence in males with 55.7 % against 42.7 % in females, whereas the intensity was 4.2–4.4. In contrast, the lowest values were recorded with *H. excavatum* for a prevalence of 13.1 % in males and 8.4 % in females and an intensity of 1.3 in both sexes (Table 2).

#### 3.2.2 Effect of camel coat colors on parasitic ticks

In terms of coat color, the sample was composed of 20 white camels, 164 brown camels (57 % of the sample) and 102 beige camels. Fig. 4 compares the number of each tick species encountered according to the dromedary's coat color*.* Regardless of tick species, camels with beige coat had an average of 0.87 ± 0.12 ticks, while those with brown and white coats averaged 1.39 ± 0.11 and 0.50 ± 0.21 ticks, respectively. *Hyalomma dromedarii* had an average of 1.54 ± 0.26 ticks for the beige coated individuals vs. 2.33 ± 0.23 ticks for the brown and 0.70 ± 0.31 ticks for the white coat color. For *H. impeltatum*, the mean of tick load was 1.02 ± 0.20 for beige, followed by brown with 1.67 ± 0.18 then white with 0.73 ± 0.53. On the other hand, the low means: 0.05 ± 0.03, 0.17 ± 0.04 and 0.05 ± 0.05 were observed for *H. excavatum* on beige, brown and white coats, respectively (Fig. 4B). The GLM revealed statistically significant differences among camel coat colors in densities of all identified species i.e., *H. dromedarii* (*p =* 0.009), *H. excavatum* (*p* = 0.008), and *H. impeltatum* (*p =* 0.032). The difference was also significant at *p* < 0.001 for densities of the three species combined.

The estimation of parasite prevalence according to coat colors revealed that brown camels were the most infested with ticks, with a prevalence of 70.1 %. Beige camels were second with 40.2 %, while white camels had the lowest prevalence with 35 %. The average intensity remained low in almost all species and varied between 4.3 and 6.5. The highest value was recorded for the beige species where it was 6.49 %. About 56.1 % of the brown camels were infested with *H. dromedarii*, while 48.2 % and 11.6 % were infested with *H. impeltatum* and *H. excavatum*, respectively (Table 2). The same pattern i.e. brown > beige > white was also observed for the abundance of tick per sampled camel.

#### 3.2.3 Variation in tick load by age of camels

Overall, the mean parasite load was 1.26 ± 0.17 ticks for age 0–2 years, 0.91 ± 0.13 ticks for 2–4 years, 1.13 ± 0.15 ticks for 4–7 years, 0.97 ± 0.18 ticks for 7–10 years, and 1.55 ± 0.24 ticks for 10–21 years. GLMs revealed that all these variations were statistically significant (*p* < 0.05), expect for *H. excavatum* that showed no significant difference (*p* > 0.311) between dromedary's age groups (Fig. 4C).

Camels in the age group of 0–2 years had an average of 2.21 ± 0.37 ticks for *H. dromedarii*, 1.44 ± 0.28 ticks for *H. impeltatum*, and 0.13 ± 0.05 ticks for *H. excavatum* (Fig. 4). These values decreased in the 2-to-4-year age group to 1.27 ± 0.24 ticks for *H. dromedarii*, 1.41 ± 0.29 ticks for *H. impeltatum* and 0.06 ± 0.03 ticks for *H. excavatum*. The variations slightly decreased or increased for the ages 4–7 and 7–10 years and stabilized in the age group of 10–21 years, recording 2.87 ± 0.52 ticks for *H. dromedarii*, 1.66 ± 0.40 ticks for *H. impeltatum* and 0.13 ± 0.07 ticks for *H. excavatum*.

On the other hand, regardless the age, the average prevalence exceeded 50 %, reaching 71 % in the oldest dromedaries with an intensity of 5.3 to 6.6. For the individual tick species, prevalence values remained high in *H. dromedarii* (35–55 %) and in *H. impeltatum* (32.5–42 %), but decreased in *H. excavatum* for all ages (4.3–10.5 %) (Table 2).

### 3.3 Extrinsic variables influencing dromedary ectoparasitism (univariate analysis)

#### 3.3.1 Numbers of ticks according to different camel farming systems

Fig. 4D shows the comparison of the number of each tick species encountered among different camel farming systems. The overall average was 1.44 ± 0.15 ticks in the extensive system, 0.35 ± 0.09 ticks in the intensive system and 1.56 ± 0.13 ticks in the mixed system. The variations of tick densities among farming systems were statistically significant for *H. dromedarii* (*p* < 0.001), *H. impeltatum* (*p* < 0.001), *H. excavatum* (*p* = 0.002), and all species pooled (*p* < 0.001). In the extensive farming system, density of *H. dromedarii* averaged 2.20 ± 0.30 ticks, while it was 0.59 ± 0.17 and 2.73 ± 0.27 ticks for the intensive and mixed systems, respectively. For *H. excavatum*, means of population densities were 0.28 ± 0.09 ticks in extensive, 0.05 ± 0.04 in intensive and 0.11 ± 0.03 for mixed systems. Finally, for *H. impeltatum*, the average was 1.84 ± 0.24 ticks in the extensive system, against 0.40 ± 0.18 ticks in the intensive system, and 1.85 ± 0.21 ticks in the mixed system.

Overall, the highest parasite prevalence (98 %) was observed in camels raised using the extensive farming system, followed by the mixed system (69.7 %) and lowest for the intensive system (16 %). The extensive farming systems was the most favorable type for ticks with a prevalence of 80 % for *H. dromedarii*, 74 % for *H. impeltatum* and low (*P%* = 18 %) for *H. excavatum*. Mixed farming system came second for all three tick species while recording the highest values for *H. dromedarii* and *H. impeltatum* with 52.8 % and 44.4 %, respectively. The intensive type of camel farming was the least affected by the different types of ticks with a prevalence of 16 % for *H. dromedarii*, 8.5 % for *H. impeltatum*, and 6.4 % for *H. excavatum*. The average intensity varied from one species to another, with scores ranging from 4.4 to 6.7 (Table 2).

#### 3.3.2 Effect of rangeland vegetation types on number of ticks

**Fig. 5:**
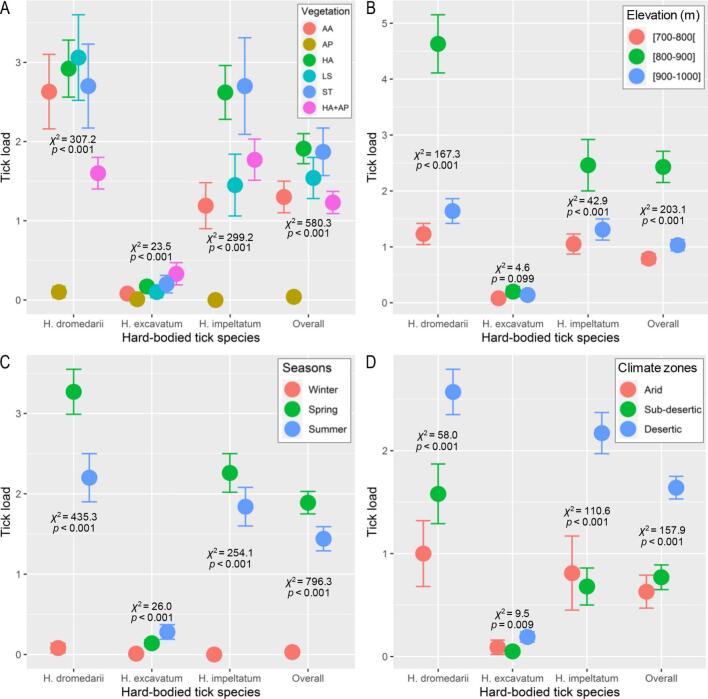
Plot of means (± standard errors, vertical error bars) of the parasite abundance of three hard-bodied tick species infesting of the one-humped camel (*Camelus dromedarius*) at different rangeland vegetation types (A), for different elevations (B), sampled seasons (C), and climate zones (D) in drylands of Algeria. The statistics *χ*^2^ and *p*-values are the summarized results of likelihood-ratio tests calculated for generalized linear model of each species separately and for all species pooled ‘overall’. (AA: *Astragalus armatus*, AP: *Aristida pungens*, HA: *Hammada articulata*, LS: *Lygeum spartum*, ST: *Stipa tenacissima*).

Fig. 5 shows the variation of the number of ticks according to different vegetation. The highest mean tick counts were highest in *H. articulata* vegetation (1.91 ± 0.19) followed by *S. tenacissima* (1.87 ± 0.30), *L. spartum* (1.54 ± 0.26), *A. armatus* (1.30 ± 0.20), and *H. articulata* + *A. pungens* (1.23 ± 0.14). The lowest densities were obtained in *A. pungens* with 0.04 ± 0.02 ticks. These variations were statistically significant (*p* < 0.001). The species *H. dromedarii* averaged 3.06 ± 0.54 in the steppe rangeland dominated by L. *spartum*, followed by *H. articulata* with 2.92 ± 0.36 ticks then *S. tenacissima* (2.70 ± 0.53 ticks) and 0.10 ± 0.07 ticks in *A. pungens* (*p* < 0.001). For *H. impeltatum*, the maximum was 2.70 ± 0.61 ticks in *S. tenacissima* and the minimum was zero in *A. pungens*. Its densities varied significantly (*p* < 0.001) between vegetation types. For *H. excavatum*, the means of tick densities were low in all rangelands with a maximum in *H. articulata* + *A. pungens* (0.33 ± 0.14) and a minimum of 0.01 ± 0.01 in *A. pungens*. These variations were also significant (*p* < 0.001).

The parasite prevalence varied greatly according to different vegetation types (Table 2). It was 100 % in the steppe rangelands of *H. articulata* + *A. pungens* followed by 96.2 % in *H. articulata*, 83.3 % in *S. tenacissima*, 67.7 % in L. *spartum*, 49.2 % in *A. armatus*, and 6.3 % in *A. pungens*. The average parasite intensity scored its maximum in *A. armatus* (7.9) against a minimum of 1.8 in *A. pungens*. The prevalence recorded high values for *H. dromedarii* and *H. impeltatum* in *H. articulata* + *A. pungens* (90 % and 76.6 %, respectively) vs. low values in *A. pungens* (*P%* = 6.3 % and 3.8 %, respectively). For *H. excavatum*, even though the values observed for parasite indices were low compared to the previous ones, the trend among vegetation remained the same.

#### 3.3.3 Number of ticks as a function of elevation

Fig. 5 illustrates the distribution of numbers of each tick species parasitizing dromedary according to site elevation. The ‘elevation’ factor revealed a statistically significant effect (*p* < 0.001) on tick numbers. This number averaged 0.79 ± 0.09 ticks in sites with 700–800 m elevation, increased to 2.43 ± 0.28 ticks at 800–900 m and then decreased to 1.03 ± 0.10 ticks at 900–1000 m. With the exception of *H. excavatum* species, the number of ticks of *H. dromedarii* (*p* < 0.001) and *H. impeltatum* species (*p* < 0.001) differed statistically significant among the three elevation ranges. Tick densities of *H. dromedarii* showed a mean of 1.23 ± 0.19 at 700–800 m that increased to 4.63 ± 0.52 at 800–900 m then drops to 1.64 ± 0.22 at 900–1000 m. Although to a lesser extent, the same trend was observed in *H. impeltatum* with a significant difference (*p* < 0.001) among elevation classes.

Overall, the prevalence increased within elevations ranging between 800 and 900 m and then decreased at 900–1000 m (Table 2). The prevalence was 42.4 % at elevation 700–800 m, increased to 89.1 % at 800–900 m and decreased to 61.1 % at 900–1000 m. The intensity was at its highest (8.2) in hosts raised at an elevation of 800–900 m. The parasite prevalence also increased with elevation for the three tick species. In *H. dromedarii*, it increased from 30.3 % at 700–800 m to 76.1 % at 800–900 m and then dropped to 50.9 % at 900–1000 m. For *H. impeltatum*, this parasitological parameter was 30.3 % at 700–800 m; it increased to 45.7 % and 43.5 at 800–900 m and 900–1000 m, respectively. The species *H. excavatum* showed a very low prevalence, increasing from 8.3 % at 700–800 m to 13 % at 800–900 m then decreased to 9.3 % at 900–1000 m. The mean intensity value varied from one species to another, with the highest values (6.1) recorded in *H. dromedarii* at 700–800 m (Table 2).

#### 3.3.4 Number of ticks by seasons

Fig. 5 compares the number of each tick species encountered according to the sampled seasons. According to GLMs, seasonal variation of tick numbers was statistically significant (*p* < 0.001), overall for all species and for each species considered separately. Overall, the mean number of ticks was highest in spring (1.89 ± 0.14) followed by summer (1.44 ± 0.15) and lowest in winter (0.03 ± 0.02). The same pattern was obtained for each species with tick density peaking in spring and summer and decreasing in winter. For *H. dromedarii*, the values recorded were: 3.27 ± 0.28 in spring, 2.20 ± 0.30 in summer and 0.08 ± 0.06 in winter. In turn, *H. impeltatum* averaged 2.26 ± 0.24 ticks in spring, 1.84 ± 0.24 in summer and lowered to zero in winter. The density values of *H. excavatum* were notably lower compared to the other two species, with counts of 0.28 ± 0.09 ticks in summer, 0.14 ± 0.04 ticks in spring, and 0.01 ± 0.01 ticks in winter.

The monitoring of parasite load revealed an increasing trend from winter to spring-summer, commencing with a very low prevalence of 1.94 % during winter. Ticks affected 80 % and 100 % of the camel population in spring and summer, respectively (Table 2). This trend was reported for all the three tick species identified, but was more pronounced for *H. dromedarii* and *H. impeltatum* (*P%* ranged between 51 and 80 %) compared to *H. excavatum* (10.5–18 %). The average intensity fluctuated between 4.4 and 6.9 with the maximum value (5.1) detected in spring for *H. dromedarii*.

#### 3.3.5 Tick load for different climate zones

Overall, the differences in the number of ticks among the climate zones (Fig. 5) was statistically significant (*p* < 0.001). The average number of ticks was 1.64 ± 0.11 in the desert climate, and then dropped to 0.77 ± 0.12 and 0.63 ± 0.16 in sub-desertic and arid climates, respectively. At the specific scale, with the exception of the *H. excavatum* species, where the variation between climates was significant at *p* = 0.009, the fluctuations of the other species densities were highly significant at *p* < 0.001. Indeed*, H. dromedarii*, recorded a mean of 1.00 ± 0.32 ticks under arid conditions against 2.57 ± 0.22 under desert and 1.58 ± 0.29 under sub-desertic climates. Similarly, for *H. impeltatum*, the average value was 2.17 ± 0.20 under the desertic climate, followed by 0.81 ± 0.36 under arid climate and 0.68 ± 0.18 under sub-desertic climate.

Parasite prevalence increased along with the increase in climate aridity (Table 2). It was 21.3 % in the arid climate, 30 % in the sub-desertic climate, and 90.7 % in the desertic climate. The prevalence index remained high for the two tick species *H. dromedarii* and *H. impeltatum* in the desertic climate, but decreased for *H. excavatum* in the same climates. The average intensity varied between 5.4 and 8.9 with a peak of 6.4 for *H. dromedarii* in the sub-desertic climate.

### 3.4 Multivariate analysis involving both intrinsic and extrinsic factors

**Fig. 6:**
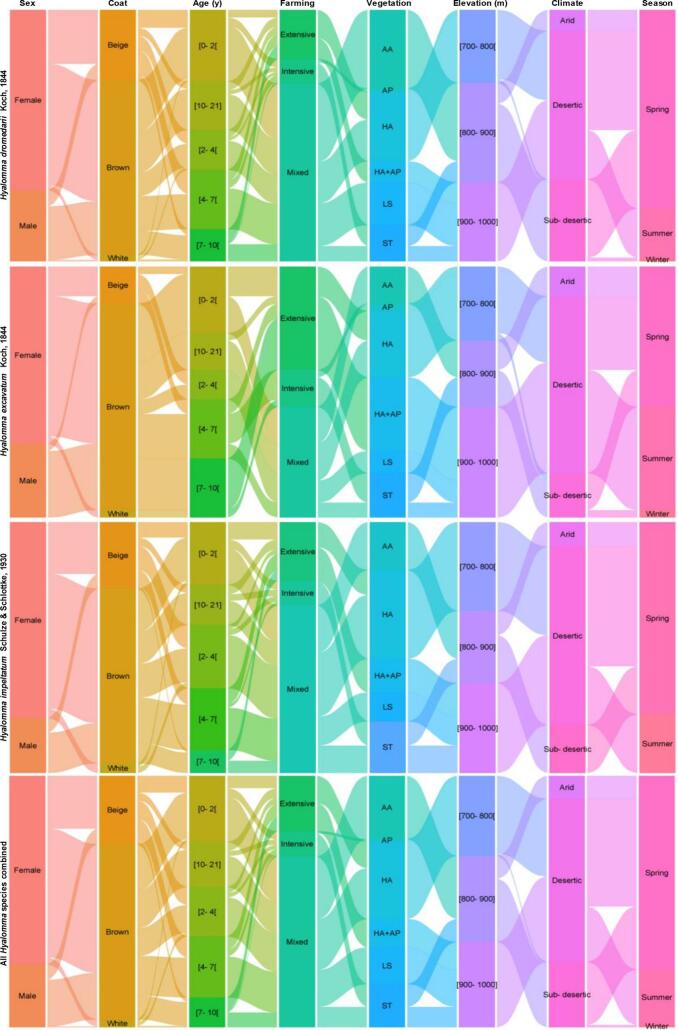
Alluvial diagrams displaying the distribution of parasite abundances of three hard-bodied tick species infesting of the one-humped camel (*Camelus dromedarius*) among different intrinsic traits (sex, coat color, and age), camel farming systems, and extrinsic factors (rangeland vegetation types, elevations, climate zones, and seasons) in drylands of Algeria. (vegetation of rangelands: AA: *Astragalus armatus*, AP: *Aristida pungens*, HA: *Hammada articulata*, LS: *Lygeum spartum*, ST: *Stipa tenacissima*).

The variation of parasite parameters and tick load was analyzed following intrinsic variables including camel sex, coat color, and, age; extrinsic factors (such as climate zones, seasons, site elevation, and steppe rangeland types of the foraging habitats), and the camel farming systems in the Sahara Desert of North Africa (Fig. 6). The generalized linear models testing the variation of tick abundances following intrinsic and extrinsic factors showed significant effects (*p* < 0.001) of rangeland vegetation and season on *H. dromedarii*, with significant tick abundance decrease in the rangelands of *Aristida pungens*, *Hammada articulata + Aristida pungens*, and *Stipa tenacissima*. Tick abundance decreased significantly during winter when compared to spring, which was not different from summer (Table 3). The GLMs revealed that tick abundances, expecially in *H. excavatum* varied significantly among seasons, expect for *H. impeltatum*. The variation of later only showed significant response to the types the vegetation, with significant abundance increase in rangelands dominated by *Hammada articulata* (*p* = 0.004) and *Stipa tenacissima* (*p* = 0.012) and decrease in *Aristida pungens* (*p* < 0.001). In the three species considered separately, the GLMs revealed that all the intrinsic variables (camel sex, coat color, and age) had no significant effect of the variation of tick abundance. However, the variation total tick abundance of these species combined showed that camel age classes, farming systems, foraging habitat elevation, and seasons had significant effects (Table 3).

**Table 3:** Generalized linear models (negative binomial distribution and log link) testing the variation of tick abundances in one-humped camel according to intrinsic and extrinsic factors in drylands of Algeria. Categories of the independent variables shown as coefficients of GLMs were selected based on the final model with the best fit (lowest AIC value) following the step-wise backward-forward selection procedure.

Variables	Estimate	Std. Error	*p*-value	Sig.
*Hyalomma dromedarii*				
Intercept 1	1.409	0.468	0.003	**
Camel sex = Male	0.151	0.197	0.441	^NS^
Camel coat color = Brown	0.020	0.194	0.917	^NS^
Camel coat color = White	−0.424	0.515	0.410	^NS^
Camel age = [2–4[	−0.017	0.269	0.950	^NS^
Camel age = [4–7[	0.442	0.258	0.087	^NS^
Camel age = [7–10[	0.100	0.288	0.727	^NS^
Camel age = [10–21]	0.124	0.274	0.652	^NS^
Farming system = Intensive	0.568	0.536	0.289	^NS^
Farming system = Mixed	0.001	0.334	0.998	^NS^
Vegetation = AP	−1.860	0.600	0.002	**
Vegetation = HA	−0.463	0.303	0.127	^NS^
Vegetation = HA + AP	−1.180	0.451	0.009	**
Vegetation = LS	−0.598	0.313	0.056	^NS^
Vegetation = ST	−0.888	0.395	0.025	*
Season = Summer	−0.221	0.392	0.680	^NS^
Season = Winter	−3.385	0.433	<0.001	***
*Hyalomma excavatum*				
Intercept 2	−1.946	0.283	<0.001	***
Season = Summer	0.673	0.473	0.155	^NS^
Season = Winter	−2.689	1.056	0.011	*
*Hyalomma impeltatum*				
Intercept 3	0.158	0.317	0.618	^NS^
Camel sex = Male	−0.178	0.261	0.497	^NS^
Camel coat color = Brown	0.229	0.253	0.365	^NS^
Camel coat color = White	−0.134	0.651	0.837	^NS^
Camel age = [2–4[	−0.208	0.317	0.512	^NS^
Camel age = [4–7[	−0.184	0.322	0.567	^NS^
Camel age = [7–10[	−0.379	0.377	0.314	^NS^
Camel age = [10–21]	0.014	0.357	0.968	^NS^
Vegetation = AP	−2.320	0.596	<0.001	***
Vegetation = HA	0.863	0.298	0.004	**
Vegetation = HA + AP	0.350	0.367	0.340	^NS^
Vegetation = LS	0.268	0.365	0.462	^NS^
Vegetation = ST	0.914	0.365	0.012	*
Overall (all species pooled]				
Intercept 4	−3.868	0.397	<0.001	***
Camel coat color = Brown	0.057	0.179	0.749	^NS^
Camel coat color = White	−0.179	0.444	0.686	^NS^
Camel age = [2–4[	0.248	0.233	0.287	^NS^
Camel age = [4–7[	0.525	0.223	0.018	*
Camel age = [7–10[	−0.139	0.262	0.595	^NS^
Camel age = [10–21]	−0.048	0.246	0.846	^NS^
Farming system = Mixed	0.678	0.274	0.013	*
Farming system = Extensive	4.299	0.422	<0.001	***
Elevation = [800–900]	0.676	0.234	0.004	**
Elevation = [900–1000]	−0.395	0.191	0.039	*
Season = Summer	−1.598	0.390	<0.001	***
Season = Winter	−2.689	0.256	<0.001	***

(*Df*: degrees of freedom, Sig.: statistical significance, ***: *p* < 0.001, **: *p* < 0.01, *: *p* < 0.05, ^NS^: *p* > 0.05)

Intercept 1: (Camel sex = Female) + (Camel coat color = Beige) + (age = [0–2]) + (Farming system = Extensive) + (Vegetation = AA) + (Season = Spring).

Intercept 2: (Season = Spring).

Intercept 3: (Camel sex = Female) + (Camel coat color = Beige) + (age = [0–2]) + (Vegetation = AA).

Intercept 4: (Camel coat color = Beige) + (age = [0–2]) + (Farming system = intensive) + (Elevation = [700–800 m]) + (Season = Spring).

Vegetation types: (AA: *Astragalus armatus*, AP: *Aristida pungens*, HA: *Hammada articulata*, LS: *Lygeum spartum*, ST: *Stipa tenacissima*).

## 4 Discussion

This study investigated the spatio-temporal variations of parasite prevalence, species richness and abundance of hard-bodied ticks in one-humped camel (*Camelus dromedarius*) in the Sahara Desert of Algeria. We identified and quantified tick species of the genus *Hyalomma* in the populations of camelids raised in three climatic zones, during three seasons, and across three camel farming systems. Our results showed that out of 980 collected ticks, three species were found: *H. dromedarii* (553 specimens), *H. impeltatum* (393 specimens) and *H. excavatum* (34 specimens). Our findings are similar to those of Seddik et al. (2016) in Tunisia, who recorded the presence of four *Hyalomma* species with a predominance of *H. dromedarii* (61 %), followed by *H. impeltatum* (22 %), *H. excavatum* (16 %) and *Hyalomma marginatum* (1 %). In Iran, Moshaverinia and Moghaddas (2015), *H. dromedarii* was similarly the predominant tick species (70.76 % of the 1122 collected ticks), followed by *H. excavatum* (19.25 %), *H. anatolicum* (4.81 %), *H. asiaticum* (4.72 %), *Rhipicephalus turanicus* (0.17 %), *H. detritum* (0.09 %), *H. impeltatum* (0.09 %) and *H. schulzei* (0.09 %). In Algeria, Bouhous et al. (2008), reported nine species: *H. dromedarii*, *H. impeltatum*, *H. impressium*, *H. detritum detritum*, *H. anatolicum anatolicum*, *H. marginatum rufipens*, *H. truncatum*, *Rhipicephalus sanguineus*, and *Rhipicephalus evertsievertsi*.

Ticks are ubiquitous and cosmopolitan species; they can infest a wide range of hosts in different ecoregions and habitats. The dromedary is the typical host of several *Hyalomma* species, especially *H. dromedarii* (Jongejan and Uilenberg, 2004). According to Dioli et al. (2001), this species is better adapted to extreme hot dry conditions (Dioli et al., 2001; Elati et al., 2018; Chandra et al., 2019). *Hyalomma impeltatum* occurs mainly in a Mediterranean steppe and desert climates. An increase in numbers of this species is observed during the spring and summer seasons. *Hyalomma anatolicum excavatum* is mainly a Mediterranean species, found in steppe or semi-desert areas and oases (Domșa et al., 2016). While cattle, sheep, goats, dromedaries, horses and donkeys are the hosts of the adults, the immature stages parasitize hedgehogs, rodents and hares (Apanaskevich and Horak, 2005; Domșa et al., 2016).

Our investigation revealed a variation in tick infestation rates among dromedaries characterized by distinct coat colors. Brown-coated camels exhibited higher tick burdens compared to lighter-coated camels, of beige or white colors. This can be explained by thermoregulation dynamics, where darker coats possibly enhance heat absorption from sunlight, creating a thermally favorable milieu for ticks whose ectothermic nature renders them responsive to temperature modulation. Enhanced warmth may escalate tick activity, growth, and persistence on brown-coated dromedaries. Furthermore, the concept of camouflage within the habitat is relevant, as the coloration of the host's coat can influence the tick's ability to locate and attach itself (Hawley and Ezenwa, 2022). The cryptic attributes of brown coats potentially render ticks inconspicuous, complicating their detection and eradication. Furthermore, ticks might possess an adaptive predisposition to affix themselves to dromedaries in desert habitats where brown coats assimilate more seamlessly. Dromedaries with brown coats might display behaviors that unintentionally increase their susceptibility to tick-prone areas, including specific grazing patterns, resting habits, or other intraspecific tendencies that expose them more to environments with a high tick presence (Rehman et al., 2017; Nicholson et al., 2019). Grooming behaviors, intrinsic to tick management, could be influenced by coat color visibility, facilitating the detection and elimination of ticks on lighter coats, while ticks on brown coats may remain inconspicuous, curtailing grooming response. Microbial communities residing on the camels' skin, contingent upon coat color, might interact with tick dynamics. Certain microorganisms could potentially compete for resources or synthesize compounds deterring tick attachment (Yu et al., 2015; Nicholson et al., 2019).

The effects of coat color maybe associated to factors like thermoregulation and grooming behavior. Darker coats (brown) might attract more ticks due to heat absorption, and ticks could be more visible on lighter coats (beige and white), promoting more effective grooming (Rehman et al., 2017). These findings collectively suggest that behavioral, physiological, and immunological factors interact to determine the observed patterns of tick infestation (Aouissi et al., 2021), contributing to our understanding of the complex dynamics between hosts and their ectoparasites (Sazmand et al., 2019; Attir et al., 2024).

Tick density correlates with the characteristics of host foraging habitats, encompassing vegetation types, hygrometry, seasonal cycles, and host diversity. The complex relationship between tick density and these factors is well-established in existing literature. Climatic elements, notably temperature and humidity, play a pivotal role in shaping vegetation, acting as significant influencers in the presence of parasites. The density and population dynamics of parasites, including ticks, are linked to these climatic variables (Idder-Ighili et al., 2015; Kadjoudj et al., 2022). The temperature-range of 7 to 10 °C represents a critical threshold for the tick life-cycle, inducing diapause characterized by reduced metabolic activity. Adverse and extreme climatic conditions contribute to decreased metabolism and delayed tick development. During winter, hard-bodied ticks adopt survival strategies, seeking refuge under leaves or in humus, where they slow down life activities as part of the diapause process (Jore et al., 2011).

The genus *Hyalomma* is a tick of the sub-desertic zone distributed between isohyets of 100–1000 mm and can only reproduce with an annual rainfall of greater than 100 mm (Bourdeau, 1993; Elati et al., 2018). *Hyalomma* spp. are rarely found at elevations higher than 1200 m and never above 1500 m (Jore et al., 2011). *Hyalomma dromedarii* is a sub-desert species that does not seem to exceed the 500 mm isohyet and should not reproduce with less than 100 mm of annual rainfall. *Hyalomma impeltatum* is typically a Sahelian tick, distributed between the 100 and 1000 mm isohyets (Camigas et al., 1986; Dieudonné et al., 2019). *Hyalomma anatolicum excavatum* is a three-host species, found in areas between the 500 and 1000 mm isohyets (Lakehal et al., 2021). This justifies its presence in the study area. In drylands, the distribution of ticks is linked to environmental factors, particularly climate and vegetation. Isohyets, representing rainfall patterns, play a vital role in determining tick presence, as ticks depend on moisture for survival. Hot desert regions, characterized by extreme heat and limited rainfall, strongly influence the productivity of natural habitats, impacting vegetation availability — the primary factor influencing tick distribution. Sparse vegetation in arid conditions can create pockets of suitable tick habitats near oases or wadis, where vegetation is more abundant (Bouallala et al., 2020; Azizi et al., 2021). This dynamic relationship is sensitive to climate change, potentially altering tick distribution and disease risk (Kovats et al., 2001; Dantas-Torres, 2015; Domșa et al., 2016).

The observed trends in associations between intrinsic variables (sex, age, and coat color) and parasitological parameters in dromedaries (Fig. 6) can be understood through biological and ecological mechanisms (Nelson et al., 2015; Nicholson et al., 2019). In terms of sex, the slightly higher tick loads in males might be attributed to behavioral differences, such as differences in grooming habits or exposure to tick habitats due to territorial behavior (Dioli et al., 2001; Hawley and Ezenwa, 2022; Miller et al., 2023). For age classes, the variation in tick loads could be linked to the development of immunity over time, where older dromedaries might have developed partial resistance to tick infestations. Younger individuals might be more susceptible due to weaker immune responses (Rehman et al., 2017; Miller et al., 2023).

In the Laghouat region of Algeria, ticks' activity period spans from March to July, with a noticeable decline in December. Spring and summer emerge as the prime seasons for heightened activity of *H. dromedarii* and *H. impeltatum*, with activity levels directly correlated to external temperature. Conversely, *H. excavatum* demonstrates significantly reduced activity during these warmer seasons. These findings align with observations in Tunisia (Khamassi Khbou et al., 2021). Nonetheless, Gray et al. (2016) reported a peak in activity for *H. excavatum* in September, which contrasts with the notably low activity levels observed throughout this study. Interestingly, Seddik et al. (2016) noted a prevalence of *H. excavatum* in autumn in Tunisia, attributed to lower temperatures. According to Qviller et al. (2014), tick species exhibit activity when daily maximum temperatures exceed 7 °C for nymphs and adults, and 10 °C for larvae; they remain largely inactive at lower temperatures or during intense heat with low humidity.

The continuous presence of these tick species (*H. dromedarii*, *H. impeltatum*, and *H. excavatum*) is likely attributed to their adaptation to arid and desert climatic conditions. These ticks can complete several cycles within a single year, contributing to their sustained presence in such environments (Walker, 2003). Ticks of the genus *Hyalomma* live in hot, arid and semi-arid biotopes, generally harsh low plains and at medium elevation, and those with long dry seasons (Lakehal et al., 2021). It is largely the most present genus in the study area. As highlighted in previous studies, ticks live in an environment and are influenced by vegetation, climatic conditions and interactions with other living things; including plants, animals, parasites and microorganisms (Dantas-Torres, 2015; Wood and Johnson, 2015).

Ticks are sensitive to climatic conditions, the temperature and hygrometry will have an important effect on their cycle and development (Viglietta et al., 2021). Nowadays winters are getting milder, which will lead to a reduction of the diapause phase and a search for the host earlier in the year. On the contrary, in summer, the population of host-seeking ticks decreases because they are on the hosts. As a result of global warming, summers are getting hotter and drier which will increase this trend and lead to an increase in the rate of development from one stage to the next. It is also important to note the importance of vegetation in the biotope of ticks (Estrada-Peña and de la Fuente, 2014). Indeed, there is a direct correlation between the presence of tick species and the type of vegetation. It is important to specify that the presence and characteristics of vegetation are reflective of the underlying climatic factors and the nature of the terrain. These factors contribute significantly to the suitability of the environment for tick development and life cycle completion (Bourdeau, 1993). Ticks typically thrive in environments with high humidity levels, which means they are commonly found in areas with abundant vegetation or a layer of dead leaves on the ground. These specific habitat requirements elucidate the presence of ticks in various ecosystems, including forests and meadows, depending on the particular humidity threshold demanded by each tick species (Sonenshine, 1991; Qviller et al., 2014; Dantas-Torres, 2015; Diuk-Wasser et al., 2021).

The extensive breeding system remains the most favorable type of parasite recruitment; we observed very high prevalence of the three ticks especially *H. dromedarii* and *H. impeltatum.* Seddik et al. (2016) found massive infestations by ticks of different species and throughout the year in a semi-extensive camel farming system. Qviller et al. (2014) demonstrated that luminosity plays a significant role in influencing ticks, as they tend to seek darker environments. Luminosity acts as a limiting factor for tick population development. High luminosity in pastures and foraging habitats leads to increased tick mortality, whereas woodlands with lower luminosity and higher air humidity provide a more favorable environment for tick development and dispersion.

## 5 Conclusions

This study, conducted for the first time in the Laghouat region of the Algerian northern Sahara, has provided valuable insights into the diversity of tick species infesting dromedaries and their geographical distribution within this region. Throughout the year, dromedaries in Laghouat were parasitized by *H. dromedarii*, *H. impeltatum*, and *H. excavatum*. Notably, this investigation highlights a preference of ticks, particularly *H. dromedarii*, for parasitizing brown-coated dromedaries, with their prevalence peaking in spring rather than in winter or summer. Furthermore, it's important to emphasize that there exists no discernible correlation between tick abundance and the age of the camels. Statistical analyses reveal that tick numbers vary significantly according to the type of farming systems and the composition of the vegetation in the habitats. Extensive and mixed farming systems appear to foster tick parasitism, while intensive farming systems exhibit a lower tick burden. Additionally, habitats dominated by vegetation like *Peganum harmala* or *Lygeum spartum* tend to host more ticks compared to those with *Aristida pungens*. These findings underscore the necessity for further comprehensive investigations to comprehensively catalog tick species across arid regions in North Africa.

The study's insights into tick infestations in dromedaries within pre-Saharan regions of Algeria hold valuable implications for their population management. By identifying tick preferences for brown-coated dromedaries, higher prevalence during spring, and variations across farming systems and rangeland vegetation types, the research enables targeted tick control and health interventions. These findings can inform optimized farming practices, livestock welfare improvements, climate-responsive strategies, and educational efforts. The potential practical applications encompass evidence-based tick management protocols, educational campaigns, selective breeding informed by genetic factors, and adapting strategies to changing climates. The study underscores the need for further research while emphasizing the broader applicability of its conclusions for arid regions, promoting sustainable camel farming practices and more effective tick control.

## Ethical approval and statement

The study was performed following ARRIVE guidelines (Animal Research: Reporting of In Vivo Experiments) and U.K. Animals Act 1986 and associated guidelines, and it was conducted according to the guidelines and standards of the Laghouat University Ethics Committee which issued the ethical approval for conducting this study project. The owners of the surveyed camels provided their verbal agreement for the tick samples to be collected after being informed about the study purpose.

## **Statement of animal rights**

All the animal studies were conducted with the utmost regard for animal welfare, and all animal rights issues were appropriately observed. No animal suffered during the course of tick sampling and collection.

## Funding

This study was not funded by any source.

## CRediT authorship contribution statement

**Rachid Chaibi:** Conceptualization, Investigation, Methodology, Resources, Writing – original draft, Writing – review & editing. **Nora Mimoune:** Conceptualization, Methodology, Writing – review & editing. **Farouk Benaceur:** Methodology, Writing – review & editing. **Latifa Stambouli:** Investigation, Resources. **Lamine Hamida:** Investigation. **Rabah Khedim:** Investigation, Methodology. **Radhwane Saidi:** Methodology, Writing – review & editing. **Mohammed Hocine Benaissa:** Formal analysis, Writing – original draft. **Hicham Gouzi:** Methodology, Writing – review & editing. **Souad Neffar:** Writing – original draft, Writing – review & editing. **Haroun Chenchouni:** Formal analysis, Visualization, Writing – original draft, Writing – review & editing.

## Declaration of competing interest

The authors declare that the research was conducted in the absence of any commercial or financial relationships that could be construed as a potential conflict of interest.

## Data Availability

The dataset used and/or analyzed during the current study is available from the corresponding author on reasonable request.
